# Computer-Aided Depth Video Stream Masking Framework for Human Body Segmentation in Depth Sensor Images

**DOI:** 10.3390/s22093531

**Published:** 2022-05-06

**Authors:** Karolis Ryselis, Tomas Blažauskas, Robertas Damaševičius, Rytis Maskeliūnas

**Affiliations:** Faculty of Informatics, Kaunas University of Technology, 44249 Kaunas, Lithuania; karolis.ryselis@ktu.edu (K.R.); tomas.blazauskas@ktu.lt (T.B.); rytis.maskeliunas@ktu.lt (R.M.)

**Keywords:** human body segmentation, depth images, image processing, point cloud

## Abstract

The identification of human activities from videos is important for many applications. For such a task, three-dimensional (3D) depth images or image sequences (videos) can be used, which represent the positioning information of the objects in a 3D scene obtained from depth sensors. This paper presents a framework to create foreground–background masks from depth images for human body segmentation. The framework can be used to speed up the manual depth image annotation process with no semantics known beforehand and can apply segmentation using a performant algorithm while the user only adjusts the parameters, or corrects the automatic segmentation results, or gives it hints by drawing a boundary of the desired object. The approach has been tested using two different datasets with a human in a real-world closed environment. The solution has provided promising results in terms of reducing the manual segmentation time from the perspective of the processing time as well as the human input time.

## 1. Introduction

Deep learning has shown great success in tackling large difficulties in several domains, including image and video processing, computer vision, and multimedia, as a key advance in artificial intelligence. It is also particularly useful for object detection, tracking, and segmentation, all of which are important tasks in recognizing human activity from images. Whole-image classification is often used for activity recognition, while pixelwise classification is often used for detection and segmentation. In this field, deep learning networks and very efficient forward and backward propagation techniques are already commonplace. Data labeling became an important part of creating a supervised neural network model. The application domains covered are likewise quite diverse [1].

For human body segmentation, three-dimensional (3D) depth images or image sequences (videos) can be used, which represent the positioning information of the objects in a 3D scene obtained from depth sensors, such as Microsoft Kinect and Intel Realsense. Despite the fact that these devices are deemed low-cost, the level of precision they attain makes them acceptable for application in a variety of research fields such as education, rehabilitation, and entertainment [2]. However, the use of low-cost depth cameras also has limitations, such as variable lighting conditions and background clutter, which can negatively affect the quality of the tracking [3]. Moreover, depth maps recorded by depth camera sensors are frequently distorted with various sorts of spatial and temporal artifacts such as Gaussian noise and hole pixels as spatial artifacts, as well as flickering between depth video frames [4]. By separating poses and removing context, the misleading context can be avoided. Furthermore, while movies in general include more information, the network may not learn the correct feature for classification (for example, learning more from the background instead of a person’s movement), causing the test case to fail [5]. Other challenges faced by depth cameras include handling fast motion and topological changes [6], which is especially relevant in the case of humans performing motions, while human body parts may become self-occluded in the process. Given a video stream of 30 frames per second and a duration of 1 min, 1800 frames have to be marked, which makes it infeasible to mark large datasets manually. However, this might be a difficult process if the network is used to mask an image and even more challenging if a video has to be masked when preparing the training dataset. Image masking is used in areas such as human body segmentation [7]. Some frequent problems, such as occlusion, distortion, motion blur, and scale variation, make object segmentation and object tracking challenging. By combining the two difficulties of video object segmentation and tracking, they can be overcome and their performance improved [8]. Human body images have regular geometric structures and layouts [9], whereas general object and scene images have significantly more complex geometric structures and layouts [10].

In this paper, a framework to assist in human body segmentation is presented with the main focus points being on the performance by minimizing both computational and human involvement over time.

The structure of the remaining parts of this article is organized as follow. Section 2 discusses the previous works on human body segmentation. Section 3 formulates the problem and describes the proposed framework. Section 4 describes the dataset and the results of the experiments. Finally, Section 5 presents the discussion and conclusions.

## 2. Related Works

In human motion analysis, segmentation of posture is critical. On a large-scale training dataset, however, high-dimensional features are memory-intensive and difficult to handle. The authors of [11] presented a two-stage dimension reduction approach (biview learning) to encode two distinct viewpoints, namely depth-difference characteristics and relative position information, in order to allow for learning a low-dimensional feature space for posture segmentation.

Depth sensor-based systems often misinterpret bodily parts, and the problem becomes more serious. This is due to the fact that applications have limited information on the correctness of recognition, and employing those parts to synthesize body postures would result in significant visual artifacts [12]. Paper [13] describes a data fusion algorithm based on algebraic operations in vector space, the system’s deployment using three depth sensor units, and the analysis of dynamic characteristics (joint position, speed of movement, functional working envelope, body asymmetry, and rate of fatigue) of human motion during physical exercise. The consistency of several elements throughout time can be used to assess a joint’s reliability, and learning the weights of dependability terms improves the performance of the classifier [14]. To capture the concurrent correlations between body joint and limb data, Huang et al. suggested a feature transfer paradigm [15]. Concurrent correlations of these features can help build a structural link that might improve the network’s inferences while reducing the requirement for refinement modules.

Human point cloud segmentation is typically challenging and difficult to achieve correct approximations, especially when there is self-occlusion and no color or texture signals. Lehment et al.’s suggested segmentation model [16] attempted to address this issue by extracting tiny regions of high saliency, such as the hands or arms, and ensuring that the information contained in these regions is not overshadowed by larger, less salient parts, such as the chest. The use of a deep recurrent hierarchical network can also provide more flexibility by minimizing or eliminating posture detection issues caused by a limited visibility human torso in the frame, also known as the frame occlusion problem, which occurs when only a portion of the body is visible [17].

Alternatively, Qin et al. [18] proposed that 3D human skeleton extraction be converted into offset vector regression and human body segmentation using a deep learning-based point cloud contraction to improve the robustness of the joint point regression.

Kulikajevas et al. pioneered an adversarial architecture [19] for point cloud recreation, built specifically for targeting humanoid shapes. The same research team suggested a three-staged deep adversarial neural network architecture for a comprehensive human body posture reconstruction capable of denoising and improving real-world depth sensor data [20], achieving impressive Earth Mover and Chamfer distances of 0.059 and 0.079. The modified approach in conjunction with the camera’s inherent settings was shown to be capable of rebuilding self-obstructed human-like morphing shapes from a depth frame [21].

Hu et al. presented an alternate approach with high operability because it is unaffected by the pose and the pose fluctuations between the two depth images, which can also reconstruct a user’s precise body shape beneath tight or loose clothing, with the added benefit of being able to generate an animatable human body model [22].

## 3. Methodology

### 3.1. Problem Statement

Say we have a depth video (a sequence of depth images)

(1) $$S=\left\{I_{0},I_{1},\ldots ,I_{n}\right\}$$

Here, *n* is the number of depth frames in the sequence and all frames are taken continuously in the same scene. We need to find binary masks

(2) $$S_{M}=\left\{M_{I0},M_{I1},\ldots ,M_{In}\right\}$$

for each frame in *S* such that values of 1 represent pixels where the desired object is present. Thus, $S_{M}$ is an image that has the same dimensions as *S* and acts as a mask for it.

$S_{M}$ could be found by a human manually marking every frame of the sequence. Because this would be a long and tedious process, a tool that helps segmenting the video quicker is required.

### 3.2. Suggested Framework

The suggested framework operates on depth streams—single stream images where each pixel’s value corresponds to its distance from the camera. Each video (image sequence) is represented as a list of 2D arrays and information about their dimensions, serialized by Protobuf [23] library. The output is a sequence of two types of images—the original image and its mask. More formally, the framework performs a transformation

(3) $$S_{M}=T\left(S\right)$$

and outputs both $S_{M}$ and *S*.

The framework can work in multiple modes:

- Full manual—the user draws the mask on the image by hand. Useful in case all other modes fail to provide the desired results. In this case, the transformation is simply user input:
  (4) $$I_{M}=H\left(I\right)$$
- Segmentation based on initial point—the user selects segmentation sensitivity *b* and a point that belongs to the desired object. Selected object is added to the mask and the process is repeated until the whole desired object is marked. In this case, the transformation is a union of autosegmented blobs of the image where initial points are provided by the user:
  (5) $$I_{M}=\cup_{i=1}^{n}A\left(P_{i},b\right)$$
- Segmentation based on the previous frame—segmentation based on initial point is performed, but initial points are taken from the previous frame. This is only applicable if at least one frame is already segmented. In this case, the transformation is the same as in segmentation based on initial point, but the points are recalculated in case the object moved:
  (6) $$I_{M}=\cup_{i=1}^{n}A\left(P_{mod}\right)$$
  where $P_{mod}$ is the recalculated point: the user-selected 2D point from the previous point cloud is taken (ignoring the depth value) and a point with the same 2D coordinates in the current frame is selected. $P_{mod}$ is found using a standard algorithm for closest point search in a kd-tree. Euclidean distance between target point and root node is calculated and the same algorithm is repeated for left and right branches of the tree recursively. If a closer point is found, the result is updated to that point. If the distance from the target point to the plane splitting the child node is greater than our best guess distance, we can eliminate the entire subtree. Selection of the new point works very well if the user selects a point near the middle of the object—even if the object moves slightly, segmentation is still applied correctly.

**Figure 1:**
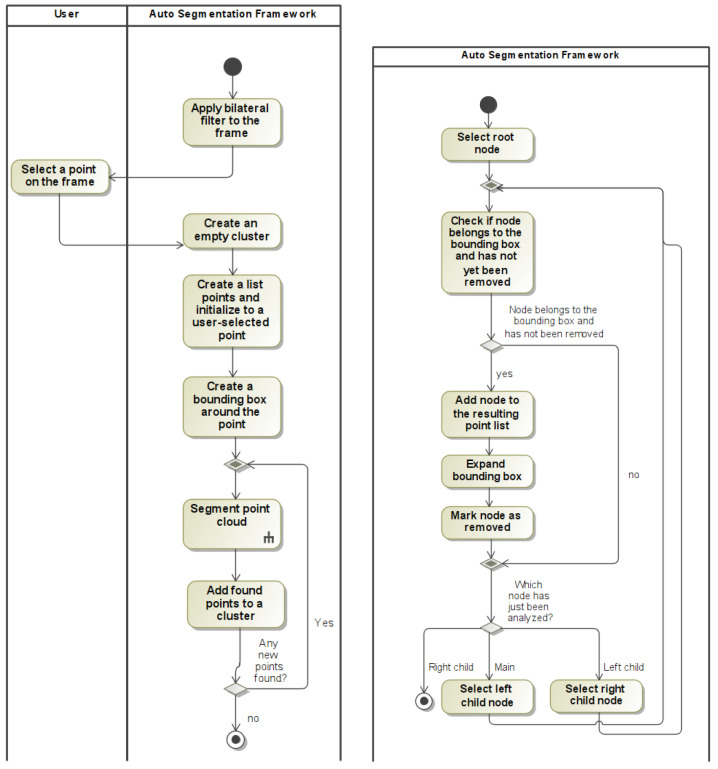
Activity diagrams: autosegmentation (**left**), segmentation inside the point cloud (**right**).

A generalized view of how the segmentation algorithm is outlined in Figure 1.

#### 3.2.1. Noise Reduction

A bilateral filter is used to reduce the noisiness of the depth frame. This filter has been chosen because it smooths the noise yet preserves object edges [24]. Because the filter blurs the image by combining values from the neighboring region of the pixel, it also smooths out the outlier points that appear in the frame because of the properties of depth cameras which makes segmenting the image easier. It was found experimentally that bilateral filter performed best with parameters $\sigma_{d}=\sigma_{r}=0.8$. Thus, the noise is reduced by applying the bilateral filter:

(7) $$I_{d}=B\left(I,0.8,0.8\right)$$

#### 3.2.2. Internal Data Representation

The depth images are stored as two-dimensional arrays of depth values. This is not an ideal frame representation because it has O(n) complexity for finding neighboring points. Because this search takes multiple passes for a single frame, after applying a bilateral filter on the frame, the depth frame is converted to a point cloud that is represented as a 3D binary tree [25]. This representation allows lower complexity of the search. Thus, after applying a bilateral filter, the frame $I_{d}$ is converted to a three-dimensional binary tree $I_{bt}$.

#### 3.2.3. Novel Clustering Algorithm

The algorithm for constructing a segment of the image defined in Formula (5) has two parameters: an initial point $P_{i}\in I_{bt}$ and a bounding box size b>0. The objective is to find such rectangular bounding box *B* that is the smallest possible bounding box containing the point $P_{i}$ and, if expanded in any dimension by *b* into a bounding box $B_{i}$, both *B* and $B_{i}$ would contain the same subset of the point cloud.

The approach used by PCL [26] (finding close points for each point separately) has a downside of having to traverse the three-dimensional tree numerous times. The point cloud is searched once for each point. Because the worst case complexity for radius search in a 3D search tree is $O(n^{\frac{2}{3}})$ [27] (*n* is the number of points in the tree), the total number of node traversals is $O(n^{\frac{5}{3}})$. If bounding boxes may be applied instead, for example, if the object in the point cloud is known to be alone in such isolated bounding box, a bounding box may be used instead of a separate sphere for each point. In a more general sense, any 3D shape may be used, but it has to fulfill one requirement: it has to have constant complexity (O(1)) to check whether a given point is inside the shape or not. A bounding box has this property because containment check involves a fixed number of comparisons. In that case, the number of traversals could be reduced. Instead of finding neighbors for each point separately, the search could be performed for a set of points. The search space would be the smallest bounding box that holds all points in the cluster expanded by *b*. After all points are found, the bounding box is recomputed and the search is repeated until no new points are added due to the expanded bounding box. This would reduce the amount of tree traversals from the number of points to the number of bounding box expansions.

In addition to that, a further optimization may be made. Instead of finding the bounding box after each search, it could be expanded during the search. This is implemented by moving the expansion of bounding box, which is a low-cost operation, inside of the tree traversing code. If the point is added to the current bounding box, the bounding box may be expanded immediately. This does not guarantee that all points are found in a single pass but reduces the number of searches inside the tree even further. Thus, the algorithm to find a single cluster for Formula (5) is shown as Algorithm 1. The novelty suggested for this algorithm is on line 28: the bounding box is expanded after adding each point to the resulting point cloud. Without this optimization, the expansion would happen after line 11.

The data structures used in the listing are:

- Bounding box, which represents a rectangular box in 3D spaces, with properties minX, maxX, minY, maxY, minZ, maxZ representing the corners of the box;
- Point, which represents a point in 3D space;
- Search tree, which has depth (0—split by x coordinate, 1—split by y, 2—split by z), location (the point that the node holds), removed (internal property to prevent returning already returned points), left and right (child nodes).

**Algorithm 1** Algorithm to find a cluster**Require:**b>0**Require:** firstPoint ∈ pointCloud
  1:
**while true do**  2:
   cluster = createEmptyCluster  3:
   currentPoints = [firstPoint]  4:
   boundingBox = point.coordinates ± b  5:
   newPointsAdded = true  6:
   **while** newPointsAdded **do**  7:
      closePoints = fcp(boundingBox,b,root)  8:
      **if** closePoints.size = 0 **then**  9:
         newPointsAdded = false10:
      **end if**11:
      currentPoints ∪= closePoints12:
   **end while**13:
   **return** cluster(currentPoints)14:
**end while**15:
**procedure** FCP(boundingBox,b,node,result=[])16:
   **if** node.depth = 0 **then**17:
      currentLocation = node.location.x18:
   **else**19:
      **if** node.depth = 1 **then**20:
         currentLocation = node.location.y21:
      **else**22:
         currentLocation = node.location.z23:
      **end if**24:
   **end if**25:
   **if** !node.removed **and** boundingBox.contains(node) **then**26:
      result ∪= node27:
      node.removed = **true**28:
      boundingBox = expand(boundingBox, node, b)29:
   **end if**30:
   points ∪= fcp(boundingBox,b,node.left,result)31:
   points ∪= fcp(boundingBox,b,node.right,result)32:
   **return** points33:
**end procedure**34:
**procedure**expand(boundingBox, node, b)35:
   boundingBox.minX = min(boundingBox.minX, node.x−b)36:
   boundingBox.maxX = min(boundingBox.maxX, node.x+b)37:
   boundingBox.minY = min(boundingBox.minY, node.y−b)38:
   boundingBox.maxY = min(boundingBox.maxY, node.y+b)39:
   boundingBox.minZ = min(boundingBox.minZ, node.z−b)40:
   boundingBox.maxZ = min(boundingBox.maxZ, node.z+b)41:
**end procedure**

In the worst case scenario, where the points are located in such a way that each bounding box expansion adds no new points, iteration count is the same as in the case of point–local sphere neighbor search. In the best case scenario, if all points are in close proximity, inside the initial bounding box, only one iteration over the tree is required. In general case, the iteration count depends on how many bounding box expansions are required to cluster the whole point cloud. The largest gain compared to the approach without autoexpanding bounding boxes is the case where every point ends up belonging to the same cluster, but the tree is constructed in such way that every point, when added, expands the bounding box. In this case, the whole tree would be added to the cluster and only 1 tree traversal would be required. The fixed bounding box approach would require 1 tree traversal for each point.

#### 3.2.4. Working with the Point Cloud Manually

The constructed point cloud, when shown to the user, is converted back to a depth image and visualized as an image where different colors correspond to different depth values. The user selects an area of the image using a square selector of selected size and the marked points are added to the selection. This is equivalent to Formula (4). The user may also mark a part of frame manually and the rest automatically.

If a segmented cluster contains too many points, they can be removed by constructing an “erased” segment. This is the same as marking a segment manually, but the cluster is explicitly excluded from the final selection.

In case the automatic or manual selection is incorrect, the user may undo the last selection. All selected clusters of points are thus stored in a stack and can be pushed and popped. In case of a completely wrong segmentation, a full clear of all the selections is available. This is equivalent to undoing all clusters.

### 3.3. Adapting the Solution to Video Streams

When a single frame is fully marked, the following frames should be similar if they come from the same video sequence. Thus, it is reasonable to try applying the same segmentation on the next frames. Because all clusters are stored separately, the same sequence of segmentation can be applied again. Each segmentation step is repeated for the next frames that are visible on the screen (1440p resolution screen fits 12 frames at once). The segmentation depends on the type of the original segmentation method.

In case of a manually marked frame, there is no semantic information about the marked area. Therefore, the only way to apply the segmentation again to mark the same area of the image. This is performed by selecting the points in the same location of the image regardless of their depth values. In case this is wrong, the user may correct the selection by erasing or marking some points. If there is an erased cluster in the original selection, the erasing is also transferred.

In case of automatic segmentation, the algorithm defined by Formula (6) is applied. User’s click location is stored and the closest point to the originally clicked point is found in the current point cloud. This acts as initial point for the automatic segmentation algorithm that is reapplied. After that, each frame can be corrected by human if needed. When the next page of frames is opened, segmentation is again transferred automatically from the last frame of the previous page. All frames of the same page are processed in parallel utilizing Java parallel streams.

#### Working with Large Video Sequences

A point cloud represented by a three-dimensional search tree proposed in this article allocates several values per point:

- Current point, consisting of 3 doubles (x, y, and z coordinates in 3D space) and 2 integers (x and y coordinates on the depth image) and a reference to it, a total of 3×8+2×4+2×8=48 bytes;
- References to the left and right child nodes of the tree. Because the application is compiled for 64-bit platforms, the references use at least 2×8=16 bytes;
- A Boolean that indicates whether the point has already been assigned to a cluster (1 byte);
- A Boolean that indicates whether the node has any child nodes that have not yet been assigned to a cluster (1 byte);
- Current dimension of the tree (an integer, 4 bytes).

This makes the total memory consumption at least 70 bytes per point. Because the resolution of a Kinect’s depth frame is 640×480 pixels and the frame rate is 30 frames per second, a 10 s video (300 frames) uses 6.2 GB of memory just to store the point clouds. This means that the tool is either limited to processing short frame sequences or the full video must not be loaded all at once. The second approach is used.

This is implemented by loading a portion of the frames into the memory, running segmentation for all loaded frames, and storing the result to disk. After that, the next portion is loaded, and the process is repeated until the full sequence is processed. Because the result is just the two frames (original depth frame and the mask) represented as flattened arrays, they take only 87 MB of memory for the same 300 frame sequence and can be loaded to the memory together and then merged.

### 3.4. Software Solution

**Figure 2:**
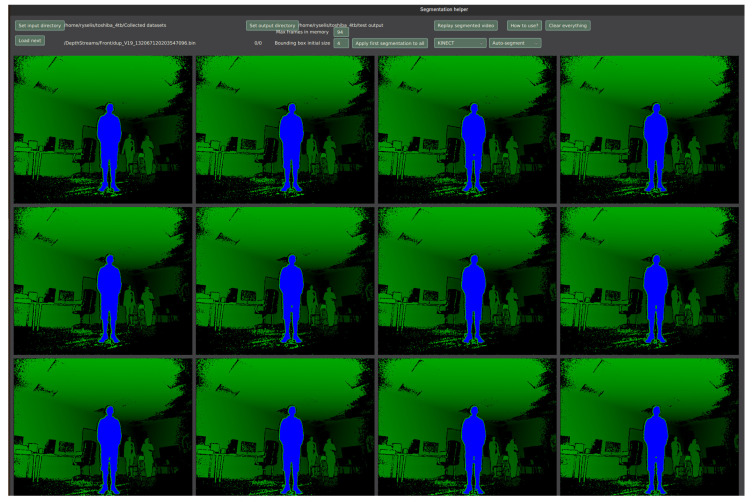
A screenshot of implemented software solution.

The implemented software solution is shown in Figure 2. A total of 12 frames are visible to the user at once (the exact number depends on screen resolution, 2560 × 1440 is used in this example). In this case, the human body was clicked in first frame and segmentation was transferred automatically to the other frames. Different shades of green represent different colors, blue represents currently selected human body mask. After the user is done with what is seen on the screen, “Next” button is clicked and the next batch of frames is loaded and immediately segmented according to the previous frames.

## 4. Framework Evaluation

### 4.1. Dataset

**Figure 3:**
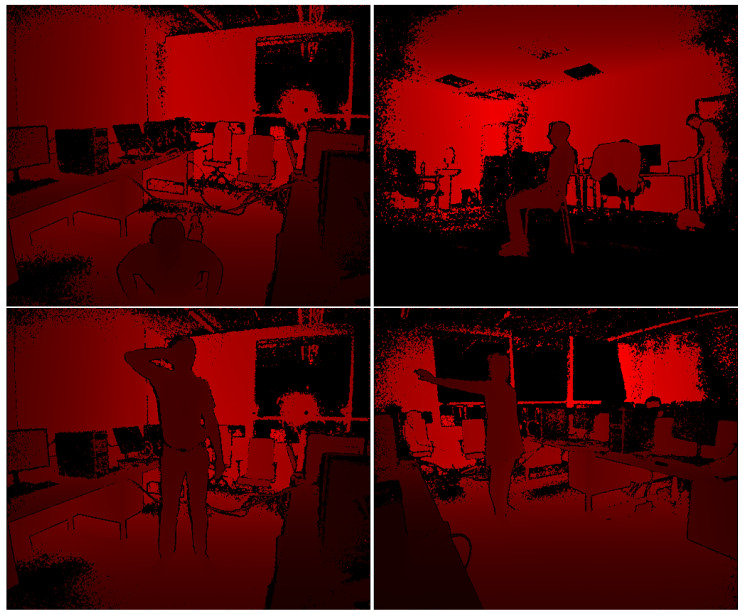
Example depth images; different colors represent different distances from the sensor.

The dataset consists of 800 depth video recordings gathered using Kinect Studio (Microsoft Corporation, Redmond, Washington, DC, USA) and custom software to convert it to a binary format using the Protobuf (Google LLC, Mountain View, CA, USA) [23] library which can then be loaded again and analyzed. The data consist of two sessions recorded with a triple Kinect setup with Kinects capturing the same room from different sides. The first dataset is recordings of people in different positions (standing with raised hands, laying on the ground, and similar) in a computer classroom with artificial light (7 people, 30 poses each, 674 recordings, over 193,000 frames). The second dataset is recordings of people either standing up or sitting on a chair in an open room with natural + artificial light (40 people, 2 poses each, 266 recordings, over 69,000 frames). There were also 295 frames captured using an Intel RealSense ZR300 (Intel Corporation, Santa Clara, CA, USA) device. Example frames are shown in Figure 3.

For our experiments, 1000 frames have been randomly selected from the two datasets containing Kinect data and 10 frames from RealSense dataset. All the images shown in Figure 3 are actually the first four frames from the benchmark dataset.

### 4.2. Test Hardware and Software

All of the benchmarks have been performed on a desktop PC running an AMD Ryzen 9 3900X CPU and 32 GB 3200 MHz DDR4 RAM (Kingston HyperX Fury Black, 2 × 16 GB on dual channel). It was tested using the Ubuntu 20.04 OS and OpenJDK Java 11.0.14.1. Custom software was implemented to run the benchmarks.

### 4.3. Performance Results

The test software has an implementation of three algorithms:

- PCL Euclidean clustering—the original PCL algorithm that uses a radius search;
- Bounding box—an algorithm that uses a bounding box search;
- Expanding bounding box—an algorithm that uses a bounding box search and expands the bounding box during the tree search (implemented in the final software solution).

All algorithms have been implemented using the Java programming language and use the same point cloud implementation. Two types of benchmarks have been performed: one for the execution time (depends on platform) and one for the tree node visit count (does not depend on platform). The PCL algorithm is implemented using the algorithm provided in their documentation and yields different results than the bounding box-based algorithms. Both bounding box-based algorithms always produce the same result given the same input. The performance benchmark runs a full segmentation of a point cloud—clusters are extracted until every point belongs to exactly one cluster.

Only the clustering time for the whole image is measured; it does not account for the tree build time which is equivalent for all of the algorithms.

The performance of the algorithms is outlined in Table 1 and Table 2. The PCL algorithm struggles with the RealSense data because it is much more noisy and the algorithm starts by yielding a large cluster (on a scale of 200k points, one third of a point cloud). Then, the algorithm has to traverse the search tree at least as many times as the number of points in the first cluster, which makes it especially slow in that case. The PCL algorithm performance is much better with the Kinect data which is generally much less noisy.

The suggested expanding bounding box algorithm is 60 times faster than the PCL segmentation and about 11 times faster than a classic bounding box method with the Kinect data. This is mainly due to the greatly reduced tree node traversal count—77 times fewer iterations over the tree compared to the PCL algorithm and almost 16 times fewer that of the classic bounding box. The worst case scenario frame is crafted to yield the same number of iterations for both bounding box algorithms.

The results show that this approach improves the classic bounding box-based search performance by a large margin and brings it closer to being able to analyze the data from depth scanning sensors without having to downscale them.

### 4.4. Fully Automatic Segmentation Accuracy Results

The full dataset has been marked using the created framework by manually checking the output of the framework and then correcting its output if it is not correct. The ground truth image masks have been obtained as the output of this image. In order to evaluate the accuracy of the algorithm, the same images have been segmented using the middle point of the human-selected point cloud that has not been added to the result cluster and the segmentation performed until either no more points could be added, or the result cluster size has reached 90% of the human-marked cluster. This has been repeated with all of the integer bounding box sizes b=1…9 and the best solution was selected because the best value *b* depends on the scene. The accuracy was calculated as

(8) $$a=\frac{n(A\cup G)}{n(A)}\times \frac{n(A\cup G)}{n(G)}$$

where *A* is a set of points marked by the segmenter, *G*-a set of ground truth points. An average of the accuracy values has been computed for each frame in the sequence.

It is important to note that even though this benchmark was performed, the goal of the solution is not to reduce the runtime while sacrificing a bit of accuracy but rather to find the middle ground between the algorithm failing so much that the user has to redo a major part of the work and being so slow that the user is waiting for too long while the data are being processed. In other words, the optimal solution is considered to be such an algorithm that minimizes the total time spent by the human. The total time consists of human manual marking or the supervision of the results and computer processing time (human wait time). However, this benchmark gives an insight on how well the algorithm performs when marking real-world data.

**Figure 4:**
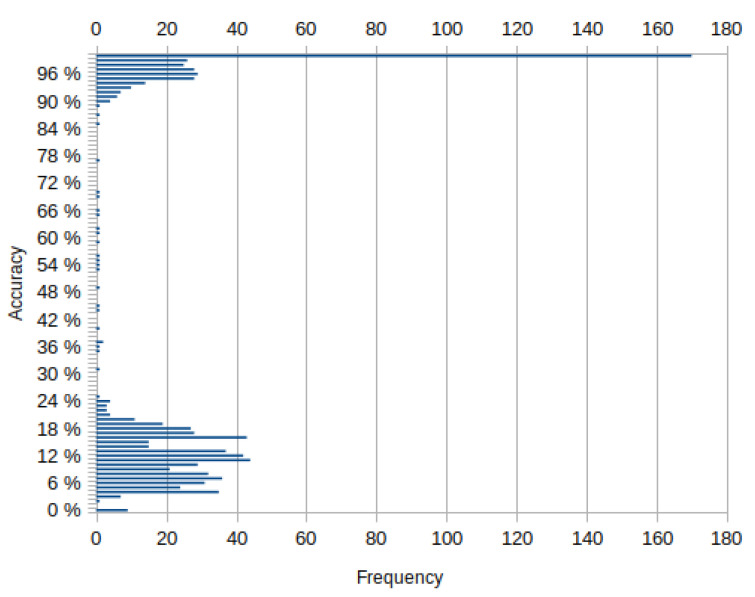
Frequencies of accuracy values.

The frequencies of the accuracy values are shown in Figure 4.

**Figure 5:**
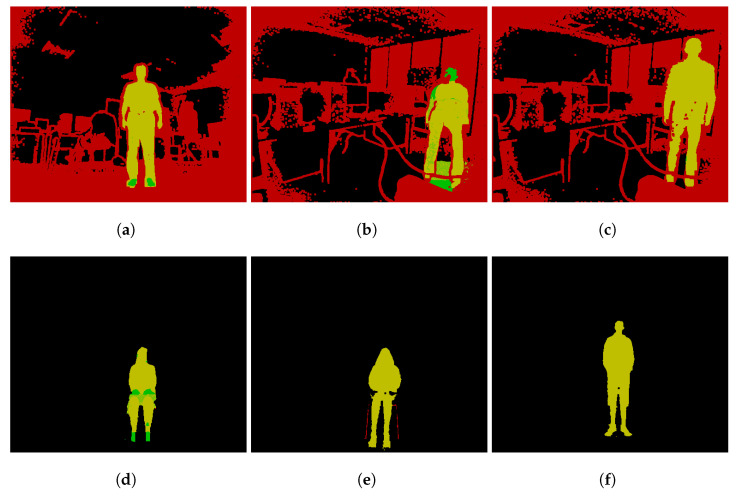
Examples of different accuracy value frames: (**a**) accuracy score 11%, (**b**) accuracy score 12%, (**c**) accuracy score 16%, (**d**) accuracy score 96%, (**e**) accuracy score 97%, (**f**) accuracy score 100%.

Most of the sequences fall either into an accuracy of above 93% or between the 4% and 20% range. The example frames of common categories are shown in Figure 5. The red color represents set *A*, green—*G*, yellow—A∪G.

The most common mistake that the segmenter makes is including some points that do not belong to the object, which expands the bounding box too much and it keeps growing indefinitely. This could be solved by the human by drawing a border manually—if it is present, it creates a cluster of points that are not added automatically which acts as an artificial wall that prevents the segmenter from selecting too many points.

The results suggest that 40% of the data could be segmented fully automatically or with very minor corrections if the user provides a correct initial point and bounding box size, while the rest required at least some user input. A total of 39.2% of all frame sequences have been segmented with an accuracy over 84%; however, this percentage is 24% for the dataset with complex poses and 76% for the dataset with simpler poses. These results confirm that the framework requires fewer human corrections for simple scenes, where the object is contained inside a clear bounding box. If the automatic segmentation fails but the segmented object edge is not moving in a part of the video, the same human-marked border can be reused which speeds up the whole manual segmentation process. The effectiveness is difficult to measure because it depends on the correct human input, but if the object is not moving and there is one bleeding edge, only that particular bleeding edge must be marked in the first frame and the framework will automatically mark the remaining video correctly. The framework does not perform well if there are many bleeding edges or the object blends with the environment. This was apparent in the videos when the human is laying on the ground.

**Figure 6:**
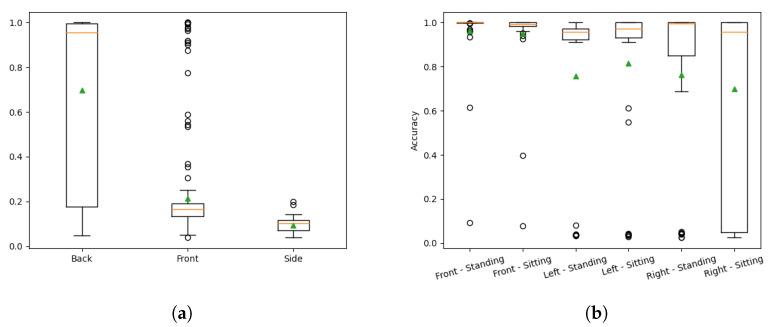
Accuracy distributions of both datasets. (**a**) Accuracy distribution of the first dataset by view side; (**b**) Accuracy distribution of the second dataset by view side and human pose.

The accuracy values for the first dataset are shown in Figure 6a. The plot is dissected by the Kinect viewing angle because there are no major differences in the accuracies, with a few exceptions. The back view camera had the best view of the human, while the side view camera usually captured a smaller human surface area, and the front camera view was occluded by a free hanging cable as seen in Figure 5b,c. The area around the human legs for the front view camera appeared noisy as well. This dataset was processed worse than the second dataset without any human supervision.

The second dataset consists of videos of humans in standing and sitting positions captured using three Kinect cameras that view the person from different sides. The accuracy values dissected by the position and the Kinect side are shown in Figure 6b. The results show that the suggested solution works well for both poses with median accuracies exceeding 99% and averages exceeding 95% with a few poor accuracy outliers. Other angles yield lower accuracies. A large portion of the frames exceeds 90% accuracy—for five out of six dissections, the first quartile is higher than at 90%.

Table 3 compares the suggested solution to other existing solutions.

Palmero et al. suggest multi-model RGB–Depth–Thermal human body segmentation [28]. The authors experiment with different techniques and obtain the best results by using random forest-based fusion. They were able to reach 79% accuracy on their own dataset which is more similar to our second dataset. However, the solution requires machine learning, and ground truth had to be marked manually. The solution is also limited to human body segmentation.

Huang et al. [29] implement a robust human body segmentation algorithm based on part appearance and spatial constraint. They create body part models and compute the probability of the pixels belonging to each part, then construct a cut-graph and segment the image based on it. The solution works with RGB images and is created specifically for human body segmentation. Li et al. also suggest a similar technique [30], but they use pictorial structures instead of body part models.

There are also CNN-based solutions that try to segment objects and can be applied to any scene. Couprie et al. perform semantic segmentation and apply it to video streams as well; however, they provide quantitative metrics only to evaluate the accuracy for still images, not for videos [31]. Wang et al. suggest their RGBD segmentation CNN which yields similar accuracy. However, both methods required pre-labeled data.

### 4.5. Manual Segmentation Time Cost Analysis

The downside of the bounding box approach is that in some cases it captures too many points and the bounding box grows more than it should. This is the reason manual segmentation is also introduced as a part of the segmentation framework. It is relatively straightforward for an experienced person to see the failing point of the bounding box approach. The user can then build a “wall” by marking one part of the human manually. For example, a common failing point is a leg with a lot of noise. Marking the leg or, depending on the case, part of the leg manually is a quick operation compared to manually marking the whole human. When this “wall” is created, the segmentation algorithm treats manually marked points as already processed and does not expand the bounding box to include them all. This manual fix enables the suggested algorithm to perform the rest of the work automatically. Moreover, if the marked part still belongs to the human body in the next frames, automatic segmentation works correctly.

The whole dataset has been marked by two people using the created framework. The exact measurements of the spent time were not made because the work was interrupted, the people were distracted. However, an approximate measure is possible by the save times of the files (accurate to the minute). The total time spent on segmenting the frame sequences, excluding frame processing, saving to disk, and other CPU-intensive tasks where the user is waiting, is almost 2 min per frame sequence or ∼0.35 s per frame.

Segmentation using the PCL algorithm has not been performed; however, the suggested framework segmented ∼40% of the data automatically, while using the PCL solution, this number is ∼70%. The human can see 12 frames on the screen at once and if the automatic segmentation is correct, it takes about 1 s for an experienced user to visually verify that the segmentation of those 12 frames is indeed correct. This means, in the case of correct segmentation, the total processing time per 12 frames is 1 s of human time + 0.016 s of processing time (the images on the screen are processed in parallel; the benchmark hardware has 24 cores so it can process all of the frames in parallel which reduces the processing time of 12 frames to approximately the processing time of 1 frame). If the PCL algorithm would be used, this would take 1 s of human time + 0.95 s of processing time. This means that the total time taken to process 40% of the data (8700 batches, 12 frames each) would be almost 2.5 h with the suggested framework and 4.7 h with the PCL solution.

It is difficult to estimate the impact of a better PCL accuracy vs. processing time; however, a rough estimate could be made. If fully automatic segmentation takes 0.08 s of user time per frame for 40%, the other part of the data should have taken 0.55 s per frame or 6.6 s per 12-frame batch to obtain the average of 0.35 s per frame. However, in cases where the PCL fails, both algorithms fail in such a way that is more difficult to fix manually because the boundaries of the object are very fuzzy. A good example of this is a human lying on the floor—the full perimeter of the human must be marked manually for both algorithms, and this is much more time consuming than just manually marking one limb. In other words, if the bounding box failure is easy to fix, the PCL would most likely not fail at all, but when it fails, a lot of the manual work must be performed. Thus, the average manual work time would most likely be higher for the worst 30% of the data. The exact number is difficult to measure, but the authors believe it could be 1.5 times higher, i.e., 9.5 s per 12 frames. If this is taken into account, the human would process 70% of the data with 1 s per 12 frames and 30% with 9.5 s per 12 frames. This reduces the human time to 0.29 s per frame rather than 0.35 s with the bounding box approach. Thus, the total processing times would be 4.5 s per 12 frames for the PCL and 4.2 s per 12 frames for the bounding box approach. This difference could save 1.8 h of total work for our datasets.

This analysis shows that the PCL algorithm may be better suited if data are noisy and the object boundaries are fuzzy, while the bounding box approach shines when the object is in an open space.

## 5. Discussion and Conclusions

Given the fact that the Kinect’s frame processing time was reduced from 184 ms to 16.3 ms while reducing the amount of node visits by 95%, the remaining 5% takes 8.8 ms. This leaves 7.5 ms for all the work that does not involve traversing the point cloud, such as collecting the results to point lists (clusters) or selecting initial points for new clusters. This shows that the most computationally expensive part of the algorithm may now take around half of the computing time.

The iteration count difference between the Kinect and the RealSense processing results shows that a good performance of the proposed algorithm is not guaranteed but provides significant improvement over classic techniques in real-life scenarios. On the other hand, even in the worst case scenarios, the expansion of the bounding box works like the non-improved version of the algorithm. This makes the improvement a viable option in all of the scenarios over the classic version. The PCL solution is much slower and is prone to a very poor performance in some cases; however, it is more accurate in most cases. The research measures the performance of the clustering of the point cloud using k-dimensional trees. The tree must be constructed before using the search. This operation may also take a portion of time which is not in the scope of this article and is therefore not considered.

The accuracy results show that the suggested framework can automatically segment a distinct object in ∼40% of the dataset. Most of the rest can be automatically segmented if the bleeding border of the object is marked manually. This can speed up the ground truth marking by an order of magnitude. If the object is isolated in space, the accuracy is usually over 92% which suggests that such datasets could be segmented by the framework only with a human as a supervisor. This is also the case with PCL algorithms; however, this sacrifices the performance. In the case of a non-cluttered depth video stream, the suggested framework is a solution that provides a lower total spent time.

## Figures and Tables

**Table 1:** Algorithm runtime comparison.

	Kinect, ms	RealSense, ms	Worst Case, ms
PCL Euclidean clustering	980	344,944	236
Bounding box	184	247	228
Expanding bounding box	16.3	45.7	145

**Table 2:** Algorithm node traverse count comparison.

	Kinect, M	RealSense, M	Worst Case, M
PCL Euclidean clustering	193.7	38,099.9	39.8
Bounding box	39.6	37.1	21.1
Expanding bounding box	2.5	4.2	21.1

**Table 3:** Comparison of state-of-the-art segmentation solutions.

Solution	Accuracy	Segments	Based on	Data Type
RGB–Depth–Thermal [28]	79%	Human	Random forest	RGBD + IR
Body part models + GC [29]	65%	Human	Geometrical + prior knowledge	RGB
Pictorial structures + GC [30]	58%	Human	Geometrical + prior knowledge	Depth
Semantic CNN [31]	65%	Any object	CNN	RGBD
Depth aware CNN [32]	49–61%	Any object	CNN	RGBD
Suggested	24–76%	Any object	Geometrical	Depth

## Data Availability

The data presented in this study are available on request from the corresponding author. The data are not publicly available due to privacy requirements.
